# The centrality of food in Norwegian adolescents’ life; a photo elicitation study among Norwegian youth

**DOI:** 10.1093/heapro/daae043

**Published:** 2024-05-29

**Authors:** Helene Aronsen-Kongerud, Sheri Bastien, Knut-Inge Klepp

**Affiliations:** Department of Public Health Sciences, Norwegian University of Life Sciences, Post Box 5003, 1432 Ås, Norway; Department of Public Health Sciences, Norwegian University of Life Sciences, Post Box 5003, 1432 Ås, Norway; Department of Community Health Sciences, Cumming School of Medicine, University of Calgary, 3D10, 3280 Hospital Drive NW Calgary, Alberta, T2N 4Z6 Canada; Department of Nutrition, Faculty of Medicine, University of Oslo, Sognsvannsveien 9, 0372 Oslo, Norway

**Keywords:** self-efficacy, adolescent, food, health, sustainability, photo elicitation

## Abstract

The purpose of the study was to explore how adolescents from a high school in Viken county define and interact with food systems in their immediate environments to understand if and how health and sustainability affect their food choices. A qualitative case study design and a participatory approach were employed. Data were collected through photo elicitation combined with group interviews. Pictures were analyzed in collaboration with participants, and the group interview through systematic text condensation. Results indicate that adolescents perceive food systems as being a substantial part of their everyday life, that they care about their health and that of the planet, and they wish to take sustainability and health into consideration when making food choices. Their food choices are affected by aspects such as family, friends, marketing, price, time, availability and accessibility. They perceive that their agency to influence their own diet and food systems is limited. Adolescents hold unique and important knowledge of their food-related behaviors and value their autonomy to make food choices. Future research and policies aiming to help adolescents make healthy and sustainable food choices should therefore actively include adolescents.

**Contribution to Health Promotion**

- Through a participatory method the study provides new knowledge of how adolescents from a high school in Viken county, Norway, define and interact with food systems.
- Results indicate that adolescents perceive food systems as being a substantial part of their everyday life.
- They care about their health and that of the planet and wish to take sustainability and health into consideration when making food choices.
- Participants’ food choices are affected by aspects such as family, friends, marketing, price, time, availability and accessibility.
- Participating adolescents perceive that their agency to influence their own diet and the food systems is limited.

## INTRODUCTION

By contributing to soil degradation, water depletion, loss of biodiversity and climate change, food systems are drivers of significant planetary changes (Poore and Nemecek, 2018; Arnold *et al*., 2021). Noncommunicable diseases (NCDs) such as cardiovascular diseases, diabetes, cancers and respiratory illnesses cause 71% of all global deaths. An unhealthy diet, promoted by current food systems, is a substantial risk factor contributing to morbidity and mortality globally (Chan, 2017; Gakidou *et al*., 2017). Food systems include all actors and activities involved in all parts of all food production chains, from production to waste management, and their associated economic, societal and natural environments (FAO, 2018). Food systems inseparably link sustainability and health. Healthy diets are associated with large portions of vegetables, fruits, whole grains, legumes, nuts and unsaturated oils, in addition to a low to moderate consumption of seafood and poultry, while limiting intake of red meat, processed meat, added sugar, refined grains and starchy vegetables (Willett *et al*., 2019). Sustainable diets have a low environmental impact and contribute to a healthy life for present and future generations (Burlingame, 2012). The characteristics of sustainable and healthy diets are not descriptive for most of the global population’s current diet (Willett *et al*., 2019).

Overweight and obesity are well-known risk factors for NCDs (NCD Risk Factor Collaboration (NCD-RisC), 2017), and the global pandemic of overweight and obesity among children and adolescents is cause for concern. Unhealthy diets contribute to these increasing rates of overweight and obesity (WHO, n.d.). Globally, adolescents’ diets are mostly affected by their immediate environments such as homes, schools, communities and their relations with others. These are in turn affected by factors such as policies, culture and economy (Fox and Timmer, 2020; Glover and Sumberg, 2020). In addition, marketing and social media are major influencers on the diet of adolescents (Caswell and Hanning, 2018; Patton *et al*., 2021; Neufeld *et al*., 2022), and marketing has been linked to unhealthy diets (Radesky *et al*., 2020). However, the interactions that take place in social media could possibly create positive health outcomes if used positively (Neufeld *et al*., 2022).

Bronfenbrenner’s socio-ecological model, exploring the reciprocal influence occurring between the systems, an individual is surrounded by, and the individual, is suitable for investigating these myriad factors (Bronfenbrenner, 1979). Intra- and interpersonal factors such as knowledge, motivation, self-efficacy (an individual’s belief in their ability to perform a certain action (Bandura, 1977)), family structure and peer support characterize the microlevel. The mesolevel describes the food and beverage environment in people’s daily life, whilst the macrolevel evolves around factors such as marketing, taxes and nutrition standards (Meltzer *et al*., 2019).

Efforts within the food systems of adolescents’ surroundings, for instance, ensuring access to nutritious and affordable foods, may support a transition to healthier diets and improved health (Canavan and Fawzi, 2019). There has been increasing attention to ensuring this transition, however, most efforts to transform the food systems have not prioritized adolescents’ increased agency (Fox and Timmer, 2020). Due to adolescents’ active participation in food systems and their unique dietary needs, interventions aiming for improved diet amongst them must be targeted at adolescents specifically (Canavan and Fawzi, 2019; Fox and Timmer, 2020). The period of adolescence, defined by WHO as aged between 10 and 19, with greater autonomy and high physical, cognitive and social development is a window of opportunity for the establishment of healthy behaviors (Flodgren *et al*., 2020). A recent systematic review of primary prevention of overweight and obesity among adolescents found that this window is often not taken advantage of. Most interventions are aimed at individual behavioral change, with few directed toward the wider community, for instance, nutrient labeling and increased prices on unhealthy foods (Flodgren *et al*., 2020). It is widely recognized that society must move away from interventions influencing individual choices, toward comprehensive policies addressing the surrounding environment (Chan, 2017; ; Flodgren *et al*., 2020). Furthermore, interventions and policies aiming to reduce the prevalence of overweight and obesity are not developed in collaboration with adolescents, even though the inclusion of their perspectives and preferences may increase the uptake of positive behaviors and adherence to policies (Flodgren *et al*., 2020; Savona *et al*., 2021).

To prevent obesity, the authentic involvement of adolescents to understand how food systems contribute to their food preferences and food-related behaviors is needed (Caswell and Hanning, 2018; Canavan and Fawzi, 2019; Browne *et al*., 2020; Koletzko *et al*., 2020). Research methods such as dialogue forums, participatory action research (PAR) and arts-based methods can ensure authentic involvement, and help adolescents become agents of change (Gold-Watts *et al*., 2021; Grewal *et al*., 2020; Johnson *et al*., 2017; Youth in CO-CREATE, n.d.). In this study, the photo elicitation method, with elements from photovoice—a method whereby photos are taken by participants and later discussed and analyzed, was implemented.

This study aims to contribute to new knowledge on three issues for which, to our knowledge, there is currently limited evidence. Firstly, it addresses the need for knowledge on how adolescents from a high school in Viken county perceive and interact with their immediate food systems. Secondly, it contributes new knowledge on the extent to which their perceptions and interactions with food systems are affected by their view on health and sustainability. Lastly, the study investigates their perceived sense of agency to influence their diet and food systems.

## METHODOLOGY

### Design

A qualitative case study design was chosen for this study (Bukve, 2016). Besides the socio-ecological model and the theory of self-efficacy, the study was guided by Ross *et al*.’s (2020) definition of agency; having the opportunity to develop self-esteem, the ability to make meaningful choices and to influence one’s political, social and material environment.

### Participants and context

Seven adolescents, four boys and three girls aged 16–18 participated in the study. They were a convenience sample from a high school situated in a community with a huge variety of food providers and a considerable agricultural production. Matvalget, an organization helping high schools to serve sustainable and healthy food in the cafeteria and free breakfast for students, aiming to establish similar food habits among adolescents, contributed to recruitment by choosing and contacting a high school within their project. In general, there are no free meals in Norwegian schools, but some communities prioritize meals in their budget. Among these communities, some collaborate with Matvalget. The school liaison helped recruit students over the age of 16, striving to achieve heterogeneity, for instance, participants of different study programs and ages. In Norway, students attend high school from 15/16 to 18/19 years of age. Therefore, the choice of conducting the study at a high school limited the age range of the study.

### Data collection

Photo elicitation, a method where photos contribute to research interviews was chosen (Harper, 2002). It was chosen to incorporate elements from photovoice due to the method’s potential to create new knowledge and to empower adolescents allowing them to become changemakers in their community (Gold-Watts *et al*., 2021). The implementation of photo elicitation and the elements incorporated from the photovoice are described below. Due to unforeseen COVID-19-related challenges, the original data collection plan was altered. Alterations are described here and discussed later. All sessions were conducted at the high school without the presence of school employees. This contributed to a safe environment where all participants were able to contribute to discussions.

In session 1, the researcher and the method were introduced, and the term ‘food system’ was discussed. The participants established their own definition of the term. This integrated definition was referred to by participants throughout the study. Moreover, participants received instructions on how to take pictures in accordance with privacy laws. The second session was conducted as a week-long homework, starting after session 1. The length of a week was chosen to provide time to take pictures and to ensure recall of information from session 1. During this session, participants used their mobile phones to take pictures of food systems in their immediate surroundings. Afterwards, they chose and uploaded four photos through the Nettskjema-picture app, an app ensuring data protection. The number of pictures was based on similar studies to ensure feasibility (Bogstad, 2018; Castellan, 2018; Flobak, 2018). The app reminded participants to take photos daily and the researcher sent three text message follow-ups. During this week, two boys withdrew from the study due to time issues.

In session 3, each participant presented their pictures based on an altered SHOWED method where the first four steps were taken advantage of; participants described what they **S**aw, what was **H**appening in the picture, how the picture was related to **O**ur (their) lives and **W**hy they believed the situation to exist (Gold-Watts *et al*., 2021). Afterwards, presentations and pictures were used as a back-drop for a discussion conducted through a semistructured group interview. A group interview was chosen to allow for discussions between participants. To avoid the negative consequences of group pressure, the sessions started with participants presenting their pictures. These presentations combined with the semistructured interview guide and the interviewer’s teaching experience created a safe environment where all opinions were appreciated. The presentations and interviews lasted for 2 h and was recorded. Due to COVID-19-related illness, two participants were unable to attend the physical interview, and an additional group interview was held on Teams, a digital platform.

In the fourth session, participants analyzed the pictures using the altered SHOWED method to categorize and describe the pictures. Participants studied the pictures before individually writing down tentative categories and descriptions on post-it notes and presenting these. Based on these presentations a discussion took place to categorize pictures and a consensus was found. This is presented in the results. COVID-19 issues further prevented three participants from participating. These were invited to a Teams-meeting but reported hectic school schedules and stress related to the schoolwork they had to catch up with because of COVID. Therefore, to not contribute to more stress, it was considered necessary to allow them to provide their input via text message. Immediately after session 4, participants helped design an exhibition of pictures, categories and descriptions in the school cafeteria. The exhibition was open for 2 weeks, allowing adolescents and employees at the school to discuss topics raised in the study.

### Analysis of group interviews

Recordings from group interviews were transcribed verbatim and pseudonyms were established for each participant. Transcripts were evaluated against recordings to ensure accurate transcription. Data from group interviews were analyzed through systematic text condensation (STC), a four-step analysis method (Malterud, 2012). First, transcripts were read, and an overview was acquired. Seven preliminary categories representing findings were established. In step 2, a table where each column represented one of the seven preliminary categories was designed. Transcripts were read several times and meaningful units were placed in the category considered fitting. During this process preliminary categories, subcategories and placement of meaningful units changed several times, as a deeper understanding of content was attained. In the third step, categories were divided into two to three subcategories by evaluating and adjusting previously established categories and subcategories. After categories and subcategories were established, a condensation of content for each subcategory was written, and each subcategory was described by one representative quote from the data. In the last step, condensations were used to write a complete analytic text. Following, titles descriptive of findings were developed for each subcategory, and transcripts were reread while actively looking for data contradictive to the results (Malterud, 2012).

### Ethical considerations

The study was approved by the Norwegian Center for Research Data. Recruitment was approved by school management who received information about the study and could ask questions before adolescents were involved. Participants received information about the study and could contact the researcher prior to and during data collection. In Norway, adolescents aged 16 can provide consent to participate in studies with nonsensitive information (NSD, n.d.), thus written informed consent was obtained from all participants prior to participation, and all participants were reminded of their opportunity to withdraw their consent at all sessions.

## RESULTS

Data analyses resulted in four categories (‘intrapersonal factors’, ‘interpersonal factors’, ‘society and policies’, ‘sustainability and health’) and nine subcategories. Three categories and seven subcategories are structured according to the socio-ecological model, whilst the category ‘sustainability and health’ and its subcategories are presented separately.

### Intrapersonal factors

#### I love being able to choose for myself, but it can be hard to do so

All participants cherish their freedom to make food choices; to buy, eat and make the food they like. The girls express needing more knowledge to be able to make healthy food choices and argue that prior food and health education was focused on performing on a test, not on enabling them to make healthy food choices. They learn about healthy food and cooking through discussions with family, friends and employees in the cafeteria, and through social media. The boys do not reflect on the need for knowledge, they feel they have knowledge of which foods are healthy. Despite this difference in perceived need for knowledge, all participants explain that they often buy food they know to be unhealthy because they feel like eating it.

#### At home I can influence, at stores not so much

Participants argue that their possibility to influence the food systems and their diet varies with different parts of the system. At home, they can influence food availability and diet within what is seen as appropriate by their caregivers. Siri explains *‘I can tell my mom and dad what I would like. Often, they will listen to me. But of course, it must be healthy. Like, I cannot have chocolate for dinner’.* Kristoffer and Hilde argue that they can influence food availability in the school cafeteria through discussions with staff or through the student council. Beyond their home and the school cafeteria participants’ possibilities to influence are limited. Lars and Hilde believe they can influence their own diet, choosing what to eat, from what is available. Influencing beyond this is difficult, for instance, influencing food availability in their surroundings. Participants believe their consumer power is limited, it does not matter what an individual does, it takes many to make a difference. Participants believe food vendors are solely interested in profit and would not listen if adolescents requested healthier food as it would yield less profit. Some participants request digital solutions for feedback, while others explain that these exist. Several participants believe taking part in this research project has increased their awareness of how to influence food systems of their surroundings.

#### Food is everything

All participants explain that they, through this research project, have become more aware of how food systems are ever-present in their lives. Food systems influence their choices, habits, health and their social life. Kristoffer argues that they are always exposed to food systems wherever they go *‘We are kind of exposed to it all the time (...) Either if we are actively seeking it or if it is like a truck from Rema 1000, or when you walk by a store on your way to something else. If you are just walking to something, you are exposed to food systems. And I believe it to be a big part of our lives because food systems are so many places’.* Hilde believes they continuously meet the food systems during their days, for instance, when one’s family eats breakfast, when classmates bring food to the classroom, when buying food in recess and when seeing marketing on social media.

### Interpersonal factors

#### Buying the food I want, where, when and with whom I want

Participants explain that food fulfills more than the physiological need to eat. Food provides a much-needed opportunity to socialize and express autonomy. After 10 years of mandatory schooling, they can finally leave school premises. The school cafeteria feels like a part of the school; therefore, it is tempting to buy food elsewhere. Furthermore, their need to socialize conquers all. The boys will not buy or eat food alone unless being assured of the possibility of socializing afterwards. Participants are affected by friends and buy the same type of food. This is not due to persuasion, but seeing others buy food makes them want the same, they end up sharing diet. Siri explains *‘I mean, I get the same diet as my friends. For instance, one of my friends and I went to this camp for four weeks. My friend was supposed to adhere to a vegetarian diet, but she ended up eating the same as I did, meat an all, because we affect each other’.*

#### My family bond around the dinner table

The girls treasure the family meal as a healthy meal and an opportunity to talk to family. They discuss how childrearing and the formation of habits during childhood affect their current food choices and argue that the restrictiveness of certain types of food in childhood makes it more tempting to buy these items now that they can. Andrea and Hilde always had access to candy during childhood. Siri grew up in a home where candy was locked away. To Hilde and Andrea, it is not important to buy candy when the possibility occurs, whilst Siri has other needs *‘Mum and dad only gave me chocolate on Saturdays (…) when I was a kid, I had to ask their permission to buy candy. Now I am free to make my own choices, I don`t have to get permission, so I just buy what I like’.* The boys do not discuss the importance of family dinners or the aspect of child-rearing. Kristoffer eats dinner with family while Lars, who lives by himself, rarely does.

### Society and policies

#### It’s all about price, availability, time and placing of food

Hilde efficiently summarizes what affects their food choices *‘And again, what matters most is price, time and availability really. How things are like presented’.* Price is a key factor affecting participants’ food choices. Bank balance, size of planned shopping and value for money influence their choices. Participants sometimes believe fast food to be the cheapest despite admitting it is not always true. They buy more food when it is on sale, in fear of missing out, not due to their longing for the product. Furthermore, time is a central influencer on their food choices. Time affects whether they choose to sleep longer or eat breakfast. It affects where they buy food. Due to their longing to leave school, they do so if time allows. Food is only bought in the cafeteria if participants are hungry and short on time. Availability and accessibility, for instance, which food outlets are close to the school and how food is placed and packed in these outlets, affect their choices. It is easiest to shop at the closest food outlet and to grab something pre-packed and placed in the check-out area. Participants argue that food outlets know this and intentionally make it easier to buy unhealthy alternatives. They argue that the only food vendor that does not exploit this knowledge is the school cafeteria, where a basket of fruit is placed in the check-out area.

#### Marketing is everywhere, and the media knows everything

The participants argue that marketing is everywhere. They explain that some marketing is easy to recognize, for instance, an advertising catalog, whilst other types of marketing are more hidden, for example, when peers bring food products with logos to the classroom. The girls are exposed to the same content in different social media and argue that this marketing is targeted. They worry about the effect of social media, trends and targeting on their food choices and diets. Moreover, they reflect on how they and their friends affect each other when sharing pictures of food. The boys do not reflect on these issues.

### Sustainability and health, does it affect my life and my food choices?

#### Maintaining my body for the rest of life, while enjoying living

Participants define health as physical and psychological and argue the importance of taking care of one’s health. The girls believe food is important to maintain health. All participants, except Hilde, believe it is hard to act according to knowledge of healthy food when buying food. Some participants argue that there is more to food than the need to take care of one’s body. Food is about socializing and enjoying life. They feel there is a conflict between enjoying life and eating healthy. Participants doubt that society will be able to help adolescents choose healthier. They argue that adolescents are diverse, that no design will fit all, and that interventions and policies must not challenge their autonomy. Despite these doubts, some participants believe that lower prices on healthy food and increased prices on unhealthy alternatives can help adolescents eat healthier. Siri, however, would buy candy even if prices increased. Hilde argues that healthy food needs to be as conveniently packed as unhealthy food to become a competitive option *‘Like a chocolate bar, it is so small, packed in plastic. You can just grab it, buy it, bring it with you, open it and eat it (…) But like apples, for instance, you must pick the apples, grab a plastic bag to put them in, put them in the bag (…)’*. Participants believe that if healthy alternatives were cheap, conveniently packed and placed in the check-out area more adolescents would buy them. However, these alternatives might not replace unhealthier alternatives but could be an addition.

#### Sustainability, so important and so difficult

Participants argue that sustainability is an important subject, covering everything from fossil fuels to food waste. Sustainability is about making resources available for generations to come. Siri explains that they must care about the environment because it is their generation who is fighting for a sustainable future. Some participants find it a bit overwhelming when their spare time and their education are filled with sustainability issues. Several participants have difficulties with choosing sustainable food alternatives, both due to a perception of having to lower one’s living standard to be sustainable, and due to a need for knowledge of which foods are sustainable. Lars describes some of the challenges he faces *‘(…) one enjoys one`s life so much, living very in the present, thinking life is so good. One does not want it to end, and one might forget what the consequences may be’.* Some participants use labels to find sustainable alternatives. They argue that it would be convenient with labels for foods that are both healthy and sustainable. Kristoffer argues that ecological food is more sustainable, but also more expensive which makes it difficult to buy. Participants explain that when it comes to food, health and sustainability are connected and both affect their food choices. However, it varies which affects them the most. Some participants believe that their participation in this research project will make them more aware of sustainability issues when making future food choices.

### Photo categories and descriptions

The photo analysis performed by the participants resulted in two categories and coherent descriptions. The first is *‘Availability, from the classroom to my hand’.* The description is*: ‘Time decides where I go. From leaving the door to having food in my hand. Who is available determines whom I go with, but I never go alone’.* The second is ‘*From earth to table’.* Its description follows: *‘From seeds, spread through transport and conscious and subconscious marketing, until it’s at the table in front of me. The choices I make are affected by the three Ps: price, placement and persons. The price, and the balance in my account, influence what and where I buy food. When I have bought what I need I can treat myself to a chocolate in the check-out area. If my friend buys buns, so do I’.*

## DISCUSSION

This study contributes new knowledge on how adolescents from a high school in Viken county perceive and interact with their immediate food systems, and to what degree their interactions are affected by their view on sustainability and health. It gives insight into their perceived agency to influence their diets and the food systems. The study is among the first Norwegian studies investigating these aspects through photo elicitation. The inclusion of elements from photovoice contributed to ensuring authentic participation and co-creation of knowledge. The main findings both with respect to the photo analysis and STC revolve around themes such as the omnipresence of food systems, socializing and food choices being affected by friends and family, marketing, time, price, availability and accessibility. The findings of this study are largely consistent with previous research which highlights the myriad factors that influence adolescents’ food-related behaviors and may be explained through the socio-ecological model (Neufeld *et al*., 2022).

Knowledge, skills, motivation, self-efficacy and attitudes are personal traits that may influence food choices (Meltzer *et al*., 2019). These intrapersonal factors affect the food choices of adolescents in this study. The girls emphasized a need for knowledge to be able to make healthy food choices. As with other European adolescents (Browne *et al*., 2020), the girls use discussions with others and online resources to learn about healthy food. Participants tend to know what the healthy alternative is and deliberately choose not to eat accordingly, highlighting the know-do gap. Some participants experience a conflict between knowing what they should eat and finally being able to make their own food choices. To them choosing the healthy alternative compromises their enjoyment of certain foods, and they are not always motivated to do so. In their review, Neufeld *et al*. (2022) found that adolescents often know which foods are healthy, while adults believe adolescents make unhealthy choices due to a lack of knowledge. This conflict, experiencing a need for knowledge whilst admitting having knowledge but not adhering to it, may express adolescents’ difficulties with choosing and eating according to knowledge. This difficulty may in part be due to adolescents being exposed to an overwhelming amount of information and may demonstrate that knowledge is necessary but insufficient in promoting dietary behavior change (Caswell and Hanning, 2018; Browne *et al*., 2020; Neufeld *et al*., 2022).

Self-efficacy can influence adolescents’ ability to adhere to a healthy diet (Meltzer *et al*., 2019; Driessen-Willems *et al*., 2021). In addition to intrapersonal factors such as motivation, perceived knowledge and skill to perform a certain action, one’s self-efficacy is affected by perceived peer support (Bandura, 1977). In this study, participants argued that unspoken social norms make it difficult to choose a diet that differs from that of one`s friends. Adolescents may not intentionally set out to prevent one another from eating healthy. However, adolescents’ diets tend to be unhealthier when with peers than at home (Bruening *et al*., 2012; Best, 2017; Browne *et al*., 2020; Fox and Timmer, 2020; Garrido-Fernández *et al*., 2020; Hermans *et al*., 2020; Neufeld *et al*., 2022). Neurodevelopmental changes occurring in adolescence increase adolescents’ need to belong with friends and family (Neufeld *et al*., 2022). This need to belong and share diet with friends could possibly affect adolescents’ perceived peer support for eating healthier, making it more difficult to eat differently from one’s friends. Participants explained that this research project made diet a conversational subject among their friends. Hopefully, this may contribute to increased perceived peer support if they wish to eat healthier.

Food systems are an influential aspect of participants’ lives providing possibilities to make autonomous choices, socialize and learn. Other studies confirm that food choices are an important part of adolescents’ identity development, providing them with an opportunity to build and express their own culture and independence, means of utmost importance when expressing agency and autonomy (Gilmour *et al*., 2020; Glover and Sumberg, 2020; Patton *et al*., 2021; Neufeld *et al*., 2022). Participants believe they have limited possibilities to influence their diet and food systems. Their agency varies with different parts of the food systems. Participants can influence food availability and diet at the microlevel, at home and in the school cafeteria. At home, participants can affect the availability of food within what is considered appropriate by their caregivers. Gilmour *et al*. (2020) found that adolescents could negotiate food within alternatives considered healthy by their caregivers. For instance, influencing which vegetables were served, not if they were served (Gilmour *et al*., 2020). Participants believe they cannot influence beyond their homes and the school cafeteria, the meso- and macrolevels of the food systems. They argue that food vendors’ number one priority is to earn money and that promoting healthy alternatives at the expense of the unhealthier ones would provide less profit. In Caswell and Hanning’s (2018) study adolescents argued the same; food vendors would lose money if they offered healthier alternatives.

Parental practices, social networks, peer pressure and support are central factors affecting food choices within the interpersonal level of the socio-ecological model (Meltzer *et al*., 2019). Participants’ food choices were affected by these aspects. Food provides an arena for socializing with friends and family, and participants rarely interact with food systems alone. Previous studies argue that shared meals hold importance for adolescents. Some adolescents choose the same food as their friends to stand in line and eat together. Other adolescents eat at fast food places because they provide an opportunity to socialize with friends without the interference of adults, a freedom not found in the school cafeteria (Best, 2017; Browne *et al*., 2020; Gilmour *et al*., 2020). Girls in this study argued the same, the school cafeteria does not provide the same freedom as food vendors outside school premises.

Participants affect and are affected by the diet of their peers. Other studies find that adolescents are often more influenced by peers than by parents and express a subconscious longing for the same food as their friends. They are easily affected by social norms and groups of friends often share the same diet. The healthiness of a group of friend’s diet is often defined by the individual (Bruening *et al*., 2012; Fox and Timmer, 2020; Garrido-Fernández *et al*., 2020; Gilmour *et al*., 2020; Hermans *et al*., 2020; Neufeld *et al*., 2022).

The girls value meals shared with family and appreciate that their diet at home is healthier than their diet at school. Several studies validate the importance family meals hold for adolescents. Adolescents associate feelings of belonging, being loved and taken care of with family meals. They appreciate how meals unite their families, providing possibilities to meet, talk and fix problems (Best, 2017; Gilmour *et al*., 2020; Simpson *et al*., 2021; Neufeld *et al*., 2022). Skipping family meals often leads to a higher consumption of unhealthy food, while eating with family provides a healthier diet and contributes to lower rates of obesity and overweight (Yuasa *et al*., 2008; Lee *et al*., 2014; Garrido-Fernández *et al*., 2020).

The girls believe conditions in upbringing influence current food choices and argue that growing up with limited access to unhealthy food may increase their longing for these foods. It is widely acknowledged that parents are vital role models affecting adolescents’ food attitudes and behaviors (Garrido-Fernández *et al*., 2020; Hermans *et al*., 2020). Regarding restriction of unhealthy foods there are conflicting results (Yee *et al*., 2017; Hermans *et al*., 2020) and concluding on the subject is outside the scope of this study. However, research suggests that the parenting style associated with restriction is a crucial factor contributing to healthy or unhealthy effects (Yee *et al*., 2017; Hermans *et al*., 2020). The overall tendency is that the availability of healthy food, restriction of unhealthy food, food rules and parental enjoyment of healthy foods may help adolescents establish a healthy diet. The authoritative parenting style, where demands are met with understanding, may have beneficial effects on diet combined with these measures. Other combinations of parenting styles and measures, such as authoritarian parenting styles with less warmth and more demands, or restriction without modeling may not have the same beneficial outcomes (Yee *et al*., 2017; Hermans *et al*., 2020). Undoubtedly, the environments in which adolescents grow up will affect their diets (Sleddens *et al*., 2011; Vollmer and Mobley, 2013; Yee *et al*., 2017; Wang and Fielding-Singh, 2018).

The meso- and macrolevels relate to the food environments where people live and factors affecting these environments such as marketing regulation, taxes and nutrition policies (Meltzer *et al*., 2019). In this and other studies, adolescents’ diet and food choices are largely influenced by convenience, expressed through availability, accessibility, desirability, affordability and time (Fox and Timmer, 2020; Gilmour *et al*., 2020). Time is essential for participants’ food choices, for instance, if they eat breakfast or sleep longer. Adolescents often skip breakfast to sleep longer (Browne *et al*., 2020; Fox and Timmer, 2020; Gilmour *et al*., 2020; Neufeld *et al*., 2022). The school’s location gives access to a wide variety of food choices, and participants only buy food in the school cafeteria if they lack time to buy food elsewhere. Adolescents’ food choices are affected by the proximity of shops, for instance, the presence of food vendors selling fast food around schools increases their consumption of these foods and decreases their consumption of healthier foods. Therefore, governments should regulate the presence of food outlets around schools (Canavan and Fawzi, 2019; Neufeld *et al*., 2022). Adolescents tend to spend their money on food and the need to experience value for money makes adolescents choose unhealthy alternatives as these are regularly on sale (Browne *et al*., 2020; Fox and Timmer, 2020; Hermans *et al*., 2020; Neufeld *et al*., 2022). Participants reported the same, unhealthy food was frequently on sale, they were afraid of missing the offer and bought more to ensure value for money. As in this study, adolescents often describe healthy food as expensive, inaccessible, unattractive and unavailable, whilst unhealthy alternatives are described as cheap, convenient, attractive and available (Riggsbee *et al*., 2019; Gilmour *et al*., 2020; Glover and Sumberg, 2020; Simpson *et al*., 2021).

Deliberate changes in choice architecture hoping to change behavior in predetermined ways without restricting alternatives or altering economic incentives are called nudging (Cesareo *et al*., 2022). Health-promoting nudges make the healthy choice easier and/or decrease the convenience of the unhealthy one (Ledderer *et al*., 2020; Song *et al*., 2021; Cesareo *et al*., 2022). School cafeterias are particularly suitable for nudging and can affect adolescents’ food choices (Cesareo *et al*., 2022). In this study, adolescents were nudged by the basket of fruit placed in the check-out area in the cafeteria. In their systematic review of nudging in school cafeterias, Cesareo *et al*. (2022) found nudges such as making healthier options more visible and accessible to have a positive effect on food choices. Another systematic review (Song *et al*., 2021) found that front-of-package labeling made people choose healthier alternatives. Importantly, 95% of studies included in this review were carried out in a laboratory setting, and results may be different in real life. Nudging can help politicians mitigate the burden of NCDs, for instance through mandatory labeling of healthy foods (Song *et al*., 2021). However, nudging has been criticized for ignoring other aspects affecting food choices such as social differences in health (Ledderer *et al*., 2020).

Participants in this and other studies feel surrounded by marketing. Combined with social, cultural and dietary norms and elements such as price, taste and convenience, marketing is a huge influencer on what adolescents prefer to eat (Caswell and Hanning, 2018; Patton *et al*., 2021; Neufeld *et al*., 2022). Exposure to marketing is associated with unhealthy behaviors, such as the intake of high-calorie, low-nutrient food and beverages (Radesky *et al*., 2020). The girls fear the effect of tailored marketing on their food choices. Targeted marketing in social media and marketing where one befriends actors, blurs the lines between advertisement, social interaction and entertainment. Currently, the trend is the marketing of less nutritious food. Despite adolescents being aware of how the marketing of unhealthy foods is more of an eye-catcher they are susceptible to these types of marketing. It contributes to their favorable attitudes toward energy-dense foods and makes social media a determinant of obesity (Caswell and Hanning, 2018; Patton *et al*., 2021; Savona *et al*., 2021; Simpson *et al*., 2021; Neufeld *et al*., 2022). On the contrary, social media could provide knowledge, peer support and guidance and help adolescents eat healthy (Neufeld *et al*., 2022). Legalization and research on marketing toward children and adolescents have not been updated according to the immense technological development. Policy measures should be put in place to ensure that children and adolescents are protected against the marketing of unhealthy food (Caswell and Hanning, 2018; Radesky *et al*., 2020; Neufeld *et al*., 2022).

Participants believe society will be unable to help adolescents adhere to a healthy diet as no intervention or policy will appeal to them all. However, they argue that changing the accessibility, presentation and availability of a product may increase consumption. They acknowledge how nudging techniques, such as labeling and placement of healthy food, may make the healthy choice easier. Economic incentives for choosing healthy food is another suggestion. Participants are unsure if these measures will make adolescents shift from unhealthy to healthy diets, or if healthy foods will be added to their current diet. Participants stress the importance of not implementing policies/interventions that reduce their autonomy. Adolescents’ unique position in life with increasing autonomy and agency, combined with their need to belong, makes it imperative that adolescents are included when interventions and policies aiming for adherence to a healthy diet among adolescents are designed (Caswell and Hanning, 2018; Canavan and Fawzi, 2019; Browne *et al*., 2020; Fox and Timmer, 2020; Simpson *et al*., 2021; Neufeld *et al*., 2022).

Adolescents in this and other studies wish to take care of their health to prevent illnesses later in life. They know that adhering to a healthy diet is important to maintain health throughout life (Gilmour *et al*., 2020; Neufeld *et al*., 2022). Simpson *et al*. (2021) found that adolescents chose the unhealthy alternatives, despite knowing what the healthy alternatives were, because they enjoyed them. This was expressed by several participants in this study as well. There are several possible reasons for this, for instance, the unconscious wish to share diet with friends, marketing and newfound autonomy.

Participants’ food choices are affected by sustainability and health. While most participants know what to eat to maintain health, they want more knowledge of sustainable foods. Several participants take advantage of food labeling when trying to choose sustainability. They request a label for food that is both healthy and sustainable. Studies argue the need to unite efforts to combat health issues for both people and planet (Caswell and Hanning, 2018; Patton *et al*., 2021). Labeling of healthy and sustainable food suggested by the EU (Bujnikci *et al*., 2020; European Commission, 2020) may help adolescents make healthy and sustainable food choices. FAO suggests incorporating sustainability issues in national dietary guidelines to enable people to make healthy and sustainable food choices (Fischer and Garnett, 2016). The inclusion of sustainability in nutrition policies could help people gain knowledge of how dietary choices, such as reducing consumption of meat and increasing that of fruit and vegetables, may be both health-promoting and sustainable (Fischer and Garnett, 2016; Oshiro *et al*., 2018). The recent Nordic Nutrition Recommendations, incorporating sustainability and health issues, therefore have a unique potential to enable adolescents to choose healthy and sustainable food.

This is a small qualitative study conducted with adolescents attending a high school situated in a highly populated county in Norway. The food systems of different Norwegian counties are diverse, ranging from urban communities to areas characterized by farming of land and sea. The high school was surrounded by food vendors and had a cafeteria available for students. Therefore, in addition to results being dependent on the characteristics of participating adolescents, results are dependent on this availability. Due to the limited age range of participating adolescents, there is a need for further studies to investigate how adolescents` actions within food systems develop during adolescence. Furthermore, COVID-19 imposed unique challenges to the data collection. It inhibited discussion between all participants, and some aspects may remain undiscussed due to this issue. Moreover, the need to evaluate picture analysis through text messages made discussion difficult and less interactive. However, the inclusive method and the safe atmosphere that was established at all sessions ensured active participation from all and provided rich data. The semistructured interview guide facilitated the investigation of unforeseen interesting aspects, such as the issue of child-rearing. Importantly, Matvalget was never in contact with the participating students and it is therefore unlikely that Matvalgets ambition of serving healthy and sustainable food has influenced the results of this study.

## CONCLUSIONS

The adolescents in this study argue that their lives are built around food systems. They want to maintain their health and that of the planet. However, despite knowing which foods are healthy, participants have difficulties adhering to this knowledge due to issues such as unspoken group pressure, longing for certain foods and newfound autonomy. They want more knowledge of what constitutes sustainable food. Participants’ diets are affected by elements across the socio-ecological model, for instance by knowledge, attitudes and self-efficacy, by peers and parents, their upbringing, access to money and to different food vendors. Diets are affected by time, convenience and accessibility, by marketing and policies. Participants experience some agency to influence their own diet, however, it is limited by what is offered, allowed and affordable. They argue that their agency to influence beyond their own diet is minimal. For instance, they cannot influence which foods are offered, where it is offered, how it is marketed and what the cost is. Future, qualitative studies are needed to further investigate how adolescents` agency to influence food systems could increase, and which interventions and policies could help adolescents maintain healthy and sustainable diets. Importantly, adolescents cherish their autonomy and hold unique knowledge of their needs. Therefore, adolescents must be actively included in future research and in the development of public health policies and interventions aiming to help adolescents adhere to a healthy and sustainable diet.

## Data Availability

The data underpinning the results of this study are not available on request.
